# Evolution of cell therapy for renal cell carcinoma

**DOI:** 10.1186/s12943-023-01911-x

**Published:** 2024-01-09

**Authors:** Yufei Wang, Eloah Rabello Suarez, Gabriella Kastrunes, Najla Santos Pacheco de Campos, Rabia Abbas, Renata Schmieder Pivetta, Nithyassree Murugan, Ghanbar Mahmoodi Chalbatani, Vincent D’Andrea, Wayne A. Marasco

**Affiliations:** 1 Department of Cancer Immunology and Virology, Dana-Farber Cancer Institute, Boston, MA 02215 USA; 2 Harvard Medical School, Boston, MA 02215 USA; 3 Center for Natural and Human Sciences, Federal University of ABC, Santo Andre, SP 09210-580 Brazil; 4 Graduate Program in Medicine - Hematology and Oncology, Federal University of Sao Paulo, São Paulo, SP 04023-062 Brazil; 5 Department of Immunology, Mayo Clinic, Scottsdale, AZ 85259 USA; 6 Department of Surgery, Brigham and Women’s Hospital, Boston, MA 02215 USA

**Keywords:** Immunotherapy, Cell therapy, Renal cell carcinoma (RCC), CAR-T, CAR-NK

## Abstract

Treatment for renal cell carcinoma (RCC) has improved dramatically over the last decade, shifting from high-dose cytokine therapy in combination with surgical resection of tumors to targeted therapy, immunotherapy, and combination therapies. However, curative treatment, particularly for advanced-stage disease, remains rare. Cell therapy as a “living drug” has achieved hematological malignancy cures with a high response rate, and significant research efforts have been made to facilitate its translation to solid tumors. Herein, we overview the cellular therapies for RCC focusing on allogeneic hematopoietic stem cell transplantation, T cell receptor gene-modified T cells, chimeric antigen receptor (CAR) T cells, CAR natural killer (NK) cells, lymphokine-activated killer (LAK) cells, γδ T cells, and dendritic cell vaccination. We have also included perspectives for using other recent approaches, such as CAR macrophages, dendritic cell-cytokine induced killer cells and regulatory CAR-T cells to shed light on preclinical development of cell therapy and advancing cell therapy into clinic to achieve cures for RCC.

## Introduction

**Fig. 1:**
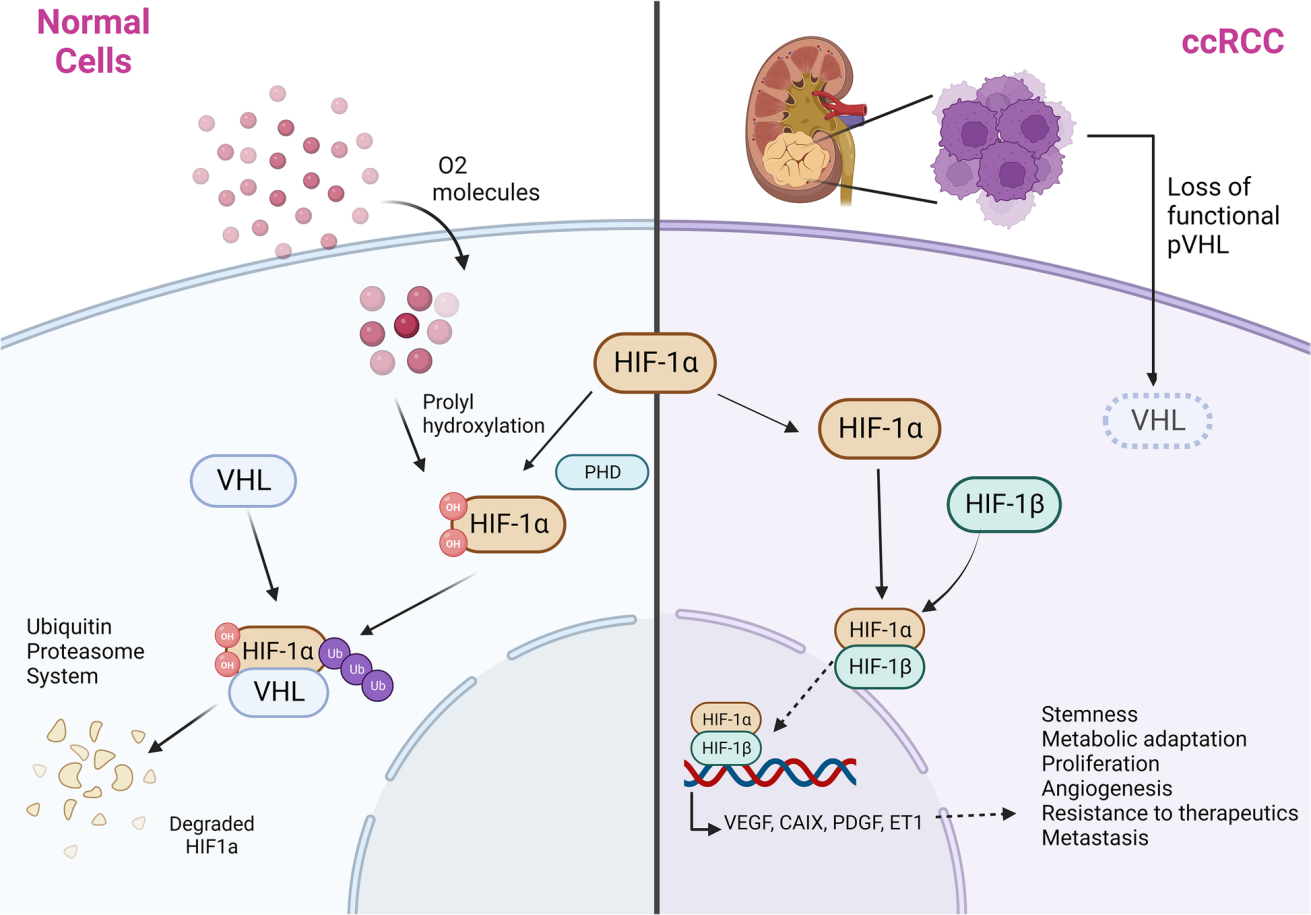
Hypoxia-inducible factor (HIF) pathway. Under normoxia, von-Hippel-Lindau (VHL) binds to HIF1α, leading to HIF1α degradation. While under hypoxia or in clear cell renal cell carcinoma (ccRCC), the dysfunction of VHL results in HIF1α-HIF1β dimer formation and HIF hyperactivation, resulting in overexpression of many downstream genes involved in angiogenesis, metabolism, and cell-cycle regulation, including carbonic anhydrase IX (CAIX), platelet-derived growth factor (PDGF) and vascular endothelial growth factor (VEGF). Created with BioRender.com

Renal cell carcinoma (RCC) represents approximately 3% of adult cancers and has been widely recognized as a heterogeneous disease encompassing different subtypes [1]. About 70–80% of RCC cases have clear cell histology (ccRCC) [1, 2], which has a relatively poor prognosis, with 30% of patients developing metastatic ccRCC. ccRCC is characterized by inactivation of the von-Hippel-Lindau (*VHL*) gene. The dysfunction of *VHL* leads to hypoxia-inducible factor (*HIF*) hyperactivation, resulting in overexpression of many downstream genes involved in angiogenesis, metabolism, and cell-cycle regulation, which represent critical therapeutic targets for patients with ccRCC [3, 4] (Fig. 1). Papillary renal cell carcinoma (pRCC) is the second most common kidney cancer, accounting for ∼15% of kidney cancers [5]. pRCC has two major types, type 1 and type 2, categorized by the presence or absence of prominent nucleoli, respectively. 80% of type 1 pRCC have an alteration in MET proto-oncogene (*MET*) genetic sequence or copy number, making *MET* a potential target pathway [6].

**Fig. 2:**
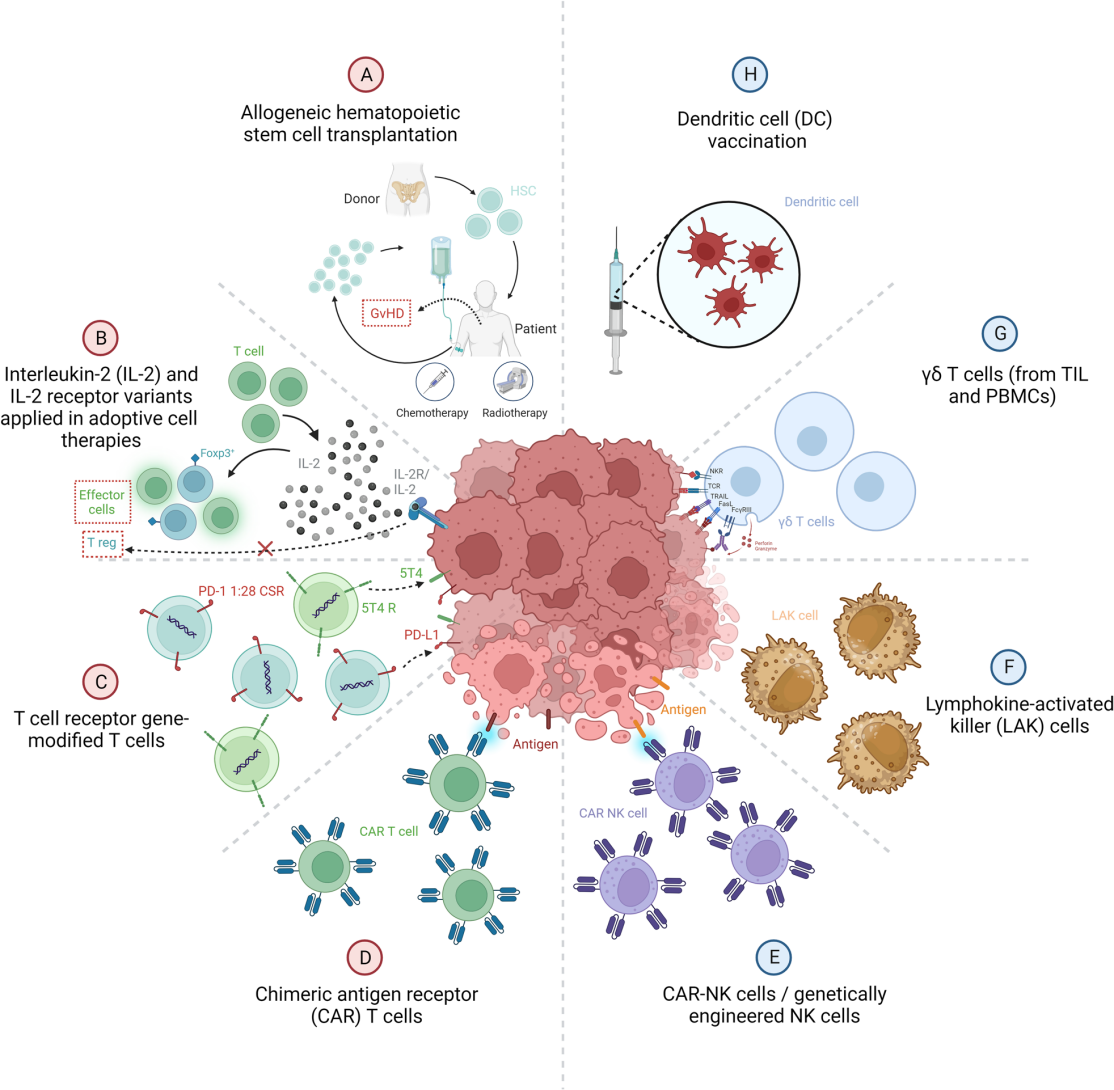
Cell therapies for renal cell carcinoma. **A** Allogeneic hematopoietic stem cell transplantation, presenting acute and chronic graft versus host disease (GvHD) with high transplant-related mortality; **B** Interleukin-2 (IL-2) and IL-2 receptor (IL-2R) variants, where mutants allowing only hIL-2Rβ activation on adoptive T cells but not the hIL-2Rα prevent T cells differentiation into Tregs and induce expansion of effector T cells against the tumor; **C** T cell receptor gene-modified T cells (TCR-T), which is consisted of a chimeric switch receptor (CSR) combining a ligand-binding domain (e.g., PD-1) with an alternative signaling domain (CD28) able to prevent T cell exhaustion and improve expansion; **D** Chimeric antigen receptor (CAR) T cells, and **E** CAR natural killer (NK) cells, both expressing engineered receptors designed against one or more antigens allowing immune cells activation against the tumor; **F** Lymphokine-activated killer (LAK) cells, that are T and NK cells, mainly expressing NK markers. Despite some efficiency against RCC metastasis, LAK cell therapy has been replaced by more specific cell-based immunotherapies; **G** γδ T cells, a subset of T cells with non-MHC‐restricted cytotoxic activity. These cells can be engineered for adoptive therapies, and the PD-1/PD-L1 axis does not abrogate their function; **H** Dendritic cell vaccination, where autologous DCs pulsed with peptides or tumor lysate-derived proteins can stimulate the generation of cytotoxic T cells in cancer patients. Created with BioRender.com

 Treatment of RCC has improved dramatically over the last decade, shifting from high-dose cytokine therapy in combination with surgical resection of tumors to extensive stage-dependent therapy regimens based on targeted therapies, highlighting the efficiency of antiangiogenic agents that targets the vascular endothelial growth factor (VEGF) pathway [7, 8] and immune checkpoint inhibitors (ICIs). In the European society for medical oncology (ESMO) clinical practice guideline, dual immunotherapy (ICI-ICI) or a combination of immunotherapy and antiangiogenic tyrosine kinase inhibitor (ICI-TKI) are recommended as the main first-line therapies for patients with advanced ccRCC. These therapies, their category, targets and comparative efficiency were summarized in Table 1 [9–11]. Despite all of these advances, curative treatment for advanced RCC remains rare [12] and the evolution of cell therapies, summarized in Fig. 2 and detailed in the text, represent a promising area for these cases. Moreover, Tables 2 and 3 summarize the results from the main pre-clinical and clinical adoptive cell therapies for RCC described below.

**Table 1:** Current targeted molecular agents recommended for the treatment of advanced/metastatic ccRCC

Name	Category	Target	Therapeutic Notes
**Pembrolizumab**	Monoclonal antibody	PD-1	First-line for advanced ccRCC when used in combination with TKI lenvatinib or axitinib. Higher PFS and OS for pembrolizumab + lenvatinib, with more serious adverse events than pembrolizumab + axitinib or nivolumab + cabozatinib [13]. Combination with lenvatinib has higher incidences of blood, lymphatic system, metabolism, and vascular disorders, while the combination with axitinib has higher incidence of cardiac and hepatobiliary toxicity, axitinib has a shorter half-life [14], usually with more easily manageable toxicities.
**Nivolumab**	Monoclonal antibody	PD-1	First-line therapy when used in combination with TKI cabozantinib or ipilimumab. Nivolumab + cabozantinib has slightly superior PFS, OS than nivolumab + ipilimumab, with more serious adverse events [13].
**Ipilimumab**	Monoclonal antibody	CTLA-4	First-line for intermediate- and poor-risk advanced ccRCC when combined with nivolumab, lower rate of severe adverse events than more potent combinations [13].
**Avelumab**	Monoclonal antibody	PD-L1	First-line for PD-L1 positive advanced ccRCC when used in combination with TKI axitinib. Avelumab is cleared faster and has a shorter half-life than other anti–PD-L1 antibodies, such as atezolizumab and durvalumab [15].
**Lenvatinib**	TKI (multi-kinase)	VEGFR, FGFR, (others)	First-line for advanced ccRCC when used in combination with pembrolizumab. Very potent TKI. Less selective. Higher PFS and OS for pembrolizumab + lenvatinib, with more serious adverse events than pembrolizumab + axitinib or nivolumab + cabozatinib [13].
**Axitinib**	TKI	VEGFR	Highly selective for VEGFR, the first line for advanced ccRCC when combined with avelumab or pembrolizumab. Better ORR, OS, and PFS compared to sunitinib [16]. Shorter half-life compared to other TKIs.
**Cabozantinib**	TKI (multi-kinase)	VEGFR (others)	First-line for advanced ccRCC when combined with nivolumab and used alone when ICI therapies are not indicated. Prolonged PFS compared with sunitinib for intermediate- or poor- risk advanced RCC [17]. Very potent TKI. Less selective. In combination with nivolumab, it has lower rates of serious adverse events compared with lenvatinib or axitinib in combination with pembrolizumab.
**Sunitinib**	TKI	VEGFR	Widely used previously as a first-line agent. Inferior performance alone compared to most first-line combinations of ICI-TKI or ICI-ICI. Option for patients for whom ICI therapy is not indicated. Superior to Sorafenib in PFS, with a similar OS [18].
**Pazopanib**	TKI	VEGFR	An alternative when ICI therapy is not indicated. Developed as a newer, more selective agent. Better patient-reported outcomes over sunitinib [19].
**Sorafenib**	TKI	VEGFR	An alternative when ICI therapy is not indicated. Inferior to sunitinib in PFS, with a similar OS. Superior to tivozanib considering OS [20].
**Tivozanib**	TKI	VEGFR	Alternative to relapsed or refractory advanced RCC following at least two prior systemic therapies. Superior PFS and ORR but inferior OS when compared to sorafenib [20].
**Everolimus**	Small molecule inhibitor	mTOR	Everolimus with lenvatinib was superior to sunitinib alone, considering PFS, without OS benefit [21] and can be recommended after first-line treatments.

*ccRCC* Clear cell renal cell carcinoma, *CTLA-4* Cytotoxic T-lymphocyte associated protein 4, *FGFR* Fibroblast growth factor receptor, *ICI* Immune checkpoint inhibitors, *mTOR* Mammalian target of rapamycin, *ORR* Overall response rate, *OS* Overall survival, *VEGFR* Vascular endothelial growth factor receptor, *PD-1* Programmed cell death receptor-1, *PD-L1* Programmed cell death ligand-1, *PFS* Progression free survival, *TKI* Tyrosine kinase inhibitor

**Table 2:** Preclinical studies of cell therapies for RCC treatment

Type	Experimental model	Conclusions	Author
TCR gene-modified T cells	**Design**: T cells transduced with 5T4-directed scFv CD3ζ receptor. **Test**: in vitro − 2220R, 2246R, 2245R cell lines.	T cells can be directed against the 5T4 protein, inducing cytotoxicity.	**Griffiths et al. 2005** [22]
	**Design**: High-avidity CD8 + T cell clones specific for an HLA-A2-restricted 5T4 epitope. **Test**: in vitro - A498, BB65, LB1828 and DOBSKI cell lines.	TCR-engineered 5T4p17-specific CD8+ T cells can induce cytotoxicity of 5T4+ RCC cell lines.	**Xu et al. 2019** [23]
	**Design**: TCR53/PD-1:28 $tm$-transgenic T cells targeting PD-L1 and PD-L2. **Test**: in vitro - RCC-26 and RCC-53 (high PD-L1 expression) cell lines.	PD-1:28 **e**ngineered T cells secreted significantly more IFN-γ, suggesting a beneficial combination with other therapeutic strategies.	**Schlenker et al. 2017** [24]
CAR-T cells	**Design**: Two humanized CAIX-directed CAR constructs: First generation Anti-CAIX G36 scFv-CD8-CD3ζ (CD8ζ) and Second-generation Anti-CAIX G36 scFv-CD28-CD3ζ (CD28ζ). **Test**: in vitro - skrc-52 (CAIX+), skrc-59 (CAIX-) cell lines in vivo* -* nude mice inoculated with the cell lines.	Second-generation G36-CD28z CAR-T cells have in vivo antitumor responses .	**Lo et al. 2014** [25]
	**Design**: Two humanized CAIX-directed CAR constructs: Anti-CAIX G36 scFv-CD28-CD3ζ (G36-CD28ζ) able to release anti-PD-L1 IgG1 or IgG4 using a bicistronic vector. **Test**: in vitro - skrc-59 (CAIX+ PD-L1+) and skrc-59 (CAIX- PD-L1-) cell lines in vivo* -* NSG mice inoculated with the cell lines.	The G36 Anti-CAIX CAR-T cells secreting human anti-PD-L1 antibodies in the ccRCC milieu boosted anti-tumor responses against RCC combating T cell exhaustion.	**Suarez et al. 2016** [26]
	**Design**: Two humanized CAIX-directed CAR construct: Anti-CAIX G36 scFv-CD28-CD3ζ (G36-CD28ζ) and Anti-CAIX G36 scFv-41BB-CD3ζ (G36-41BBζ) able to release anti-PD-L1 IgG4 using a bicistronic vector. **Test**: in vitro – skrc-59 (CAIX+ PD-L1+) and skrc-59 (CAIX- PD-L1-) cell lines in vivo* - NSG mice inoculated with the cell lines.*	Anti-CAIX G36 CD28 CAR-T cells releasing anti-PD-L1 IgG4 antibodies offered exciting new prospects for the treatment of refractory ccRCC and hypoxic tumors.	**Campos et al. 2022** [27]
	**Design**: Comparison of second-generation humanized anti-CAIX G36- scFv CD28- CD3ζ and anti-CAIX G36-41BB CD3ζ) with a third-generation CAR (anti-CAIX G36-CD28-41BB- CD3ζ) using different CD4/CD8 proportions. **Test**: in vitro - skrc-59 (CAIX+) cell line. in vivo* -* NSG-SGM3 mice inoculated with the cell lines.	Anti-CAIX BBζ CAR4/8 CAR-T cells have the potential to be translated to clinic for treatment of ccRCC due to complete tumor regression.	**Wang et al. 2021** [28]
	**Design: **CAIX-directed CAR-T cells composed by a mouse anti-human CAIX-scFv, 4-1BB-CD3 ζ CAR in combination with the TKI sunitinib. **Test**: in vitro - Ketr-3 and OSRC-2 cell lines. in vivo* -* NOG mice inoculated with the cell lines.	Combination therapy with CAIX-CAR-T and sunitinib showed synergistic efficacy in a mouse lung metastasis model of human RCC.	**Li et al. 2020** [29]
	**Design**: PARPi olaparib (OLA) associated with CD70 directed-CAR-T 4-1BB CD3ζ. **Test**: in vitro - 786-0, A498, and 769-P cell lines. in vivo* -* NDG mice inoculated with 786-0 cell line.	This study indicates that the combination of CAR-T cell therapy with PARPi represents a potential therapeutic approach for RCC.	**Ji et al. 2021** [30]
	**Design**: Analysis of multiple anti-CD70 scFvs 4-1BB CD3ζ allogeneic CAR-T cells associated with ritumixab. **Test**: in vitro - 786-0 (CD70+++), ACHN (CD70++) and REH (CD70+) cell lines. in vivo* -* NSG mice inoculated with the 786-0 cell line.	These efficacy data supported the evaluation of CD70 CAR-T cells for the treatment of RCC and has led to the advancement of an allogeneic CD70 CAR-T cell candidate into Phase I clinical trials.	**Panowski et al. 2022** [31]
	**Design: **c-met-directed third generation CAR-T cells containing CD28, 4-1BB and CD3ζ, in combination with axitinib. **Test**: in vitro - A498 (c-met+++) and KMS11 cell line. in vivo* -* NSG mice inoculated with A498-Luc cell line.	This study demonstrated the potential of anti-c-met CAR-T cell alone or in association with axitinib against RCC.	**Mori et al. 2021** [32]
CAR-NK cells	**Design**: CAR based on anti HER2 scFv FRP5-CD28- CD3ζ (CAR 5.28.ζ ). **Test**: in vitro - Murine Renca-lacZ/HER2, Renca-lacZ/EGFR cells lines. in vivo* -* NSG implanted with Renca-lacZ/HER2 cell line.	NK-92/5.28ζ has antitumor activity resulting in fewer lung metastases compared to control.	**Schonfeld et al. 2014** [33]
	**Design**: Third-generation CAR‑NK92 cells (anti-CAIX scFv-CD28-41BB-CD3ζ) alone or combined with bortezomib. **Test**: in vitro - OSRC-2, Ketr-3, ACHN and 293 cell lines. in vivo* -* NSG mice implanted with Ketr-3luc+ cell line.	CAIX-Specific NK92 cells alone decreased RCC volume and the association with bortezomib boosted their antitumor effects, leading to complete remission in mice.	**Zhang et al. 2018** [34]
	**Design**: Third-generation CAR based in an EGFR-scFv-CD28-4-1BB-CD3ζ associated with cabozantinib. **Test**: in vitro - 786-O, ACHN, Ketr-3 and OSRC-2 cell lines. in vivo* -* ACHN-Luc implanted in NSG mice.	EGFR-directed CAR-NK92 cells decreased EGFR+ RCC, and the association with cabozantinib boosted the antitumor effects of the anti-EGFR CAR-NK92 cells.	**Zhang et al. 2017** [35]
Genetic engineered NK cells	**Design**: NK cells transduced with human CXCR2**.** **Test**: in vitro - ACHN, Caki-2, A498 cell lines.	CXCR2-transduced NK cells showed increased adhesion properties and ability to migrate along a CXCR2 ligands gradient.	**Kremer et al. 2017** [36]
γδ T cells	**Design**: IL-15-induced γδ T cell compared with IL-2-γδ T cell. **Test**: in vitro - 786-O, ACHN, Caki-1 cell lines. in vivo* -* Patient-derived xenografts, NOG mice.	These results indicate that γδ T cells induced by IL-15 are more potent against RCC compared to IL-2-induced γδ T cells.	**Zhang et al. 2021** [37]
	**Design**: CD3 low Vγ9+-δ1+ TILs and peripheral blood Vγ9+-δ2 + T cells. **Test**: in vitro - Caki-1 and ACHN cell lines.	Vγ9Vδ1 T cells induced cytotoxicity of RCC cells.	**Lee et al. 2021** [38]
CAR-T cells and oncolytic adenovírus	**Design**: Oncolytic adenovirus carrying decorin (OAV-DEC) with a mouse anti-human CAIX scFv-CAR-T 4-1BB-CD3ζ. **Test**: in vitro - OSRC-2 (CAIX+++), 786-0 (CAIX++) and ACHN (CAIX+) cell lines. in vivo* -* NSG mice using the OSRC-2 cell line.	Combined use of OAV-Decorin and CAIX targeted CAR-T displayed synergistic antitumor effects in vitro and in vivo by enhancing T cell persistence.	**Zhang et al. 2022** [39]

*RCC* Renal cell carcinoma, *5T4* Trophoblast glycoprotein, *TCR* T-cell receptor, *E:T* Effector to target rate, *CAIX* Carbonic anhydrase IX, *LAK* Lymphokine activated killer cells, *PD-L1* Programmed cell death ligand-1, *CAR-T* Chimeric antigen receptor T cell, *IFN-γ* Interferon-gamma, *IL* Interleukin, *KO* Knockout, *ATT* Adoptive T-cell therapy, *HER-2* Epidermal growth factor 2 receptor, *EGFR* Epidermal growth factor receptor, *NK* Natural killer cell, *NGFR* Nerve growth factor receptor, *PBMC* Peripheral blood mononuclear cell, *CXCR2* Chemokine (C-X-C motif) receptor 2, *scFv* Single chain variable fragment

**Table 3:** Clinical studies of cell therapies for RCC treatment

Phase	Clinical design	Clinical response	Author
I	**Treatment**: CD70-targeting allogeneic CAR-T cell therapy (CTX-130™). **Protocol**: Standard lymphodepleting chemotherapy with fludarabine 30 mg/m² and cyclophosphamide 500 mg/m² for 3 days, followed by CTX-130™ infusion at dose levels ranging from 3 × 10^7^ to 9 × 10^8^.	7.7% durable CR (18 + months) and 69.2% SD (4 months).	**Pal et al. 2022** [40]
I	**Treatment**: Autologous T cells genetically retargeted against CAIX CD4 gamma-based CAR-T cells. **Protocol**: T cells expressing scFvG250-CD4 gamma receptor. Cohort 1: Inpatient up titration; max 8 IV infusions with 2 × 10^7^ a 2 × 10^9^ T cells each in two cycles, combined with 5 × 10^5^ IU/m^2^ human rIL-2 SC, twice a day at days 1–10 and 17–26. Cohort 2: 3 × 3 Phase I approach, start dose: 1 × 10^8^ T cells/ infusion at a maximum of 10 infusions in two cycles; combined with 5 × 10^5^ IU/m^2^ human rIL-2 SC, twice a day at days 1–10 and 29–38. Cohort 3: same as cohort 2 plus pre-treatment with 5 mg anti-CAIX mAb G250 IV infusion 3 days before start each series of T cell infusions.	12 patients with no clinical responses recorded.	**Lamers et al. 2006, 2013** [41, 42]
I/II	**Treatment**: DC vaccination. **Protocol**: Antigen-pulsed autologous dendritic cells. Intranodal or intradermal vaccinations at a 1 × 10^7^ dose (at least 6 vaccinations): 4 vaccinations: 1/27; 6 vaccinations: 14/27; 7–27 vaccinations: 12/27. Second vaccination was combined with 2 × 10^6^ IU IL-2 SC (days 2–6) and 18/27 patients received 5% imiquimod topical (2–6 hs before vaccination).	Response after 8 weeks: 13/27 SD and 14/27 PD. Median overall survival: 16.6 months.	**Berntsen et al. 2008** [43]
I	**Treatment**: LANAK cells associated with IL-2 infusions. **Protocol**: IL-2 Cycles at 1-week intervals (at a daily dose of 16-20 × 10^6^ IU/m^2^ infused alone for 48 h), apheresis 2 days after the end of each course, followed by reinfusion of LANAK cells (prepared in vitro) 2 weeks after apheresis + a 3-day cycle of 16-20 × 10^6^ IU/m^2^IL-2 daily.	3/10 PR, 4/10 CR and 1/10 SD with immunotherapy alone, and 2/10 CR after immunotherapy plus surgery.	**Escudier et al. 1994** [44]
I	**Treatment**: Autologous cell-therapy product based on γ9δ2 T cells (Innacell γδ™). **Protocol**: 3 cycles of 1-h intravenous infusion of γ9δ2 T cells with 3-week intervals (doses from 1 up to 8 × 10^9^ cells), combined with IL-2 SC (2 × 10^6^ IU/m^2^ from DAY 1 to 7) in the second and third cycles.	6/10 SD, 4/10 PD and 25.7 weeks median time to progression.	**Bennouna et al. 2008** [45]
II	**Treatment**: LAK cells with human rIL-2. **Protocol**: 48/94 continuous infusion arm group: rIL-2 1 mg/m²/d IV infusion. LAK cells administered IV on days 11,12 and 14. 46/94 bolus injection group: rIL-2 33 µg/kg IV bolus infusion. LAK cells administered IV on days 11, 12 and 14. Both groups: one course of treatment every 3 months (3 courses total).	Bolus injection: 3/46 CR and 6/46 PR. Continuous infusion: 2/48 CR and 5/48 PR.	**Weiss et al. 1992** [46]
I	**Treatment**: Autologous CIK cells in combination with DCs and tyrosine kinase TKIs **Protocol**: Group 1: sunitinib/sorafenib monotherapy; Group 2: DCs + auto-CIKs + sunitinib/sorafenib	Significantly higher median PFS (28.0 vs. 11.0 months); 3-year OS rate (57.1% vs. 28.6%); more SD (11/15 vs. 6/19); less PD (8/15 vs. 9/19), and death (3 vs. 5) in group 2 compared to group 1.	**Mai et al. 2018** [47]
I	**Treatment**: Autologous CIK cells in combination with DCs and pembrolizumab (anti PD-1) **Protocol**: 31 patients assessed for response to treatment with DCs + auto-CIKs + pembrolizumab. All patients enrolled in trial were given the therapeutic.	2/31 CR, 5/31 PR, 13/31 SD and 11/31 PD (1/8 CR, 1/8 PR, 4/8 SD, 2/8 PD in RCC); Overall disease control rate is 64.5%	**Chen et al. 2018** [48]

*RCC* Renal cell carcinoma, *PD* Progressive disease, *SD* Stable disease, *PR* Partial response, *CR* Complete response, *GvHD* Graft-versus-host disease, *CRS* Cytokine release syndrome, *GR* Grade, *CAIX* Carbonic anhydrase IX, *scFv* Single-chain variable fragment, *IV* Intravenous, *SC* Subcutaneous, *mAb* Monoclonal antibody, *rIL-2* Recombinant interleukin-2, *LANAK* Lymphokine-activated natural killer, *CIK* Cytokine-induced killer cells, *TKI* Tyrosine kinase inhibitors, *LAK* Lymphokine-activated killer cells, *DC* Dendritic cells

## Preclinical and clinical applications of adoptive cell therapies for RCC: acquired knowledge and perspectives

### Allogeneic hematopoietic stem cell transplantation

Allogenic hematopoietic stem cell (HSC) transplantation, named allo-HSCT, has succeeded against primary and metastatic RCC due to an immune graft-versus-tumor (GvT) effect [49]; however, graft-versus-host disease (GvHD) challenges its application. Bregni et al. summarized the findings of 14 clinical studies on HSC transplantation for RCC patients, showing response rates ranging from 0 to 71% [49]. In half of the patients, acute and chronic GvHD were present, with transplant-related mortality observed in 0–33% of patients [49, 50]. These two post-transplant events, GvT and GvHD, are considered “two faces of the same coin” [51]. In the treatment of leukemia, administration of short-term immunosuppressive agents, camphorsulfonic acid (CsA) or methotrexate (MTX) at 10 mg/week, post allo-HSCT has been shown to significantly reduce GvHD without an appreciable impact on the GvT [52], which is promising to be translated to tame the immune overactivation in RCC treatment.

Alkylating agents alone or with total body irradiation (TBI) are the most used agents for myeloablative conditioning in clinical allo-HSCT. However, they are very cytotoxic and frequently induce and amplify GvHD. Some recent alternative approaches have been explored in a review paper by Saha and Blazar [53]. The use of radioimmunoconjugates in the conditioning has shown clinical benefits, with decreased relapse and no changes in transplant-related mortality. One interesting strategy allowed transplantation tolerance to fully major histocompatibility complex (MHC) mismatched donor marrow using nonmyeloablative preconditioning with busulfan or irradiation with lower systemic doses combined with costimulatory pathway blockade and/or immunosuppressive drugs. In preclinical setting, the use of antibodies and immunoconjugates as preconditioning methods for allo-HSCT have been tested, with the advantage of more specific targeting of hematopoietic stem and immune cells, reducing global toxicity. These antibodies can be used alone in high doses or in lower doses associated with traditional conditioning agents in reduced doses, inducing lower toxicity compared with the traditional methods and achieving comparable allo-engraftment [53].

### Interleukin-2 (IL-2) and IL-2 receptor variants applied in adoptive cell therapies

Despite the importance of IL-2 against metastatic cancer [54], this cytokine has various limitations, including its dual action on regulatory T cells (Tregs) and effector T cells. IL-2 can induce CD4+ CD25+ Foxp3+ Treg expansion, especially in the immunosuppressive ICOS+ population [55]. In 2021, Motzer et al. engineered an orthogonal IL-2/IL-2R pair that do not cross react with their wild-type counterparts to specifically activate adoptively transferred T cells [56]. This method only activates the hIL-2Rβ on adoptive T cells but not the hIL-2Rα (CD25), preventing T cells differentiation into Tregs, thus ameliorating complications associated with conventional IL-2 therapy. Several IL-2 fusion proteins and variants are showing considerable promise in pre-clinical and clinical trials. PD-1-IL-2v, a new immunocytokine that binds PD-1 and IL-2Rβγ in cis, recovers the ability to differentiate PD-1+ T cell factor 1 (TCF-1)+ stem-like CD8+ T cells [57], which are critical for the success of PD-1 blockade based immunotherapies [58–61]. Another fusion protein, ALKS-4230, consisting of IL-2 and the extracellular domain of IL-2Rα, inhibiting interaction with IL-2Rα and preferentially binding to IL-2Rβγ [62, 63], is currently being investigated in Phase I/II clinical trial (NCT02799095). GI-102 has a CD80-ectodomain N terminal and IL-2Rβ targeted C terminal domain, is being tested in Phase I/II trials (NCT05824975) as a single agent. An IL-2 mutant (F42A, Y45A and L72G), fused to an anti-fibroblast activation protein mAb 4B9 [64], named FAP-IL-2v (simlukafusp alfa) is in Phase I/II clinical trials for RCC and other various carcinomas through combination therapies with trastuzumab, cetuximab, bevacizumab, and pembrolizumab (NCT02627274, NCT03193190, NCT03063762, NCT03386721, and NCT03875079). Cergutuzumab, sharing the same IL-2 mutein, fused with a carcinoembryonic antigen (CEA) targeted antibody [65], is also being tested in the clinic as a single therapeutic (NCT02004106), or in combination with atezolizumab (NCT02350673) for the treatment of metastatic solid tumors.

### T cell receptor gene-modified T cells

#### T cell receptor-engineered T cells targeting 5T4 Tumor antigen

Trophoblast glycoprotein (TPBG, 5T4) is a heavily N-glycosylated antigen, a member of the family of proteins containing leucine-rich repeats (LRRs). The elevated prevalence of this protein is seen in human trophoblasts and across many primary and metastatic cancers, and its expression is restricted in adult systemic tissues [66]. The overexpression of 5T4 in ovarian, gastric, pancreatic, renal, and colorectal malignancies has been associated with poor prognosis and reduced OS of patients [22, 67–70]. Griffiths et al. have shown 5T4 positivity on three RCC cell lines (2220R, 2245R, and 2246R) and 20 RCC patient samples [22]. This study also proved that 5T4-targeted T cells could elicit cytotoxic activity on RCC tumor cells, paving the way for exploring T cell-based therapeutic strategies targeting 5T4-enriched tumors. A first line of 5T4 redirected CD8+ T cells selectively eliminated 5T4+ kidney, breast, and colorectal cancer cells in vitro [23].

#### Chimeric PD-1:28 receptor

**Fig. 3:**
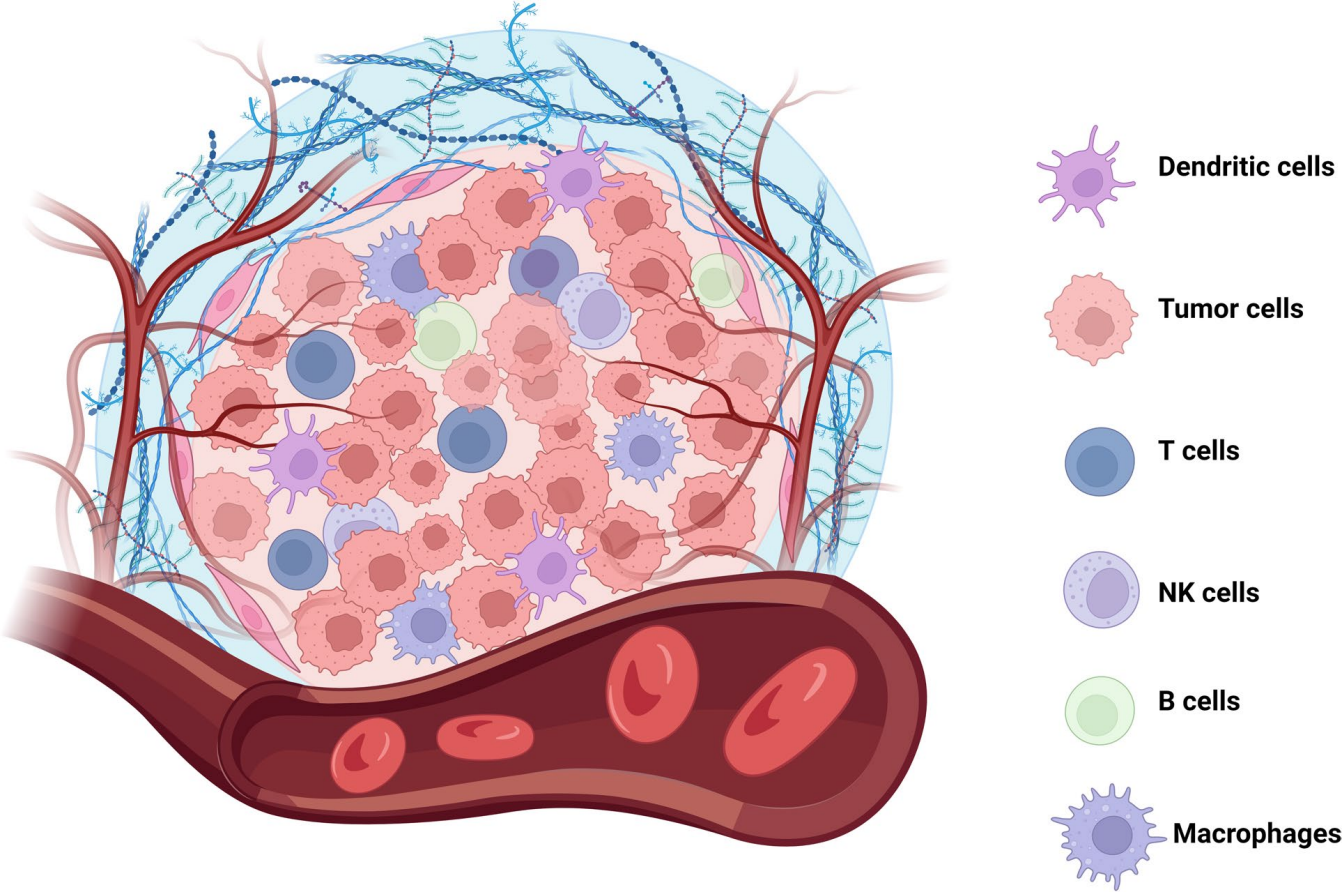
Immunosuppressive tumor microenvironment (TME). The TME is comprised of tumor cells, stroma, and exhausted immune cells, including dendritic cells (DCs), T cells, NK cells, B cells and macrophages. Created with BioRender.com

One of the main obstacles to achieving a durable antitumor response with T-cell-mediated therapy is the exhaustion/inactivation of the T cells due to immunosuppressive factors in the tumor microenvironment (TME) [71] (Fig. 3). Multiple workarounds for this problem have been proposed and executed, including constructing CAR-T cells with various payloads to prevent or revert T cell exhaustion. A chimeric switch receptor (CSR) combines a ligand-binding domain with an alternative signaling domain. Several such constructs have been used for enhancing and altering signaling pathways in adoptive cell therapy (ACT) [72–75]. The PD-1:28 CSR is a fusion of the extracellular domain of PD-1 and the signaling domain of the costimulatory receptor CD28. In highly immunosuppressive TMEs, activation through the costimulatory domain has been shown to prevent T cell exhaustion and improve expansion [76]. At the same time, blocking immune checkpoint signals such as the PD-1/PD-L1 interaction is crucial to avoid T cell inactivation and restore effector function [77]. The PD-1:28 CSR restored effector functions essential for tumor cytotoxicity, preventing Th2 polarization and blocked PD-1 function in cell lines derived from various malignancies, including RCC, squamous cell carcinoma, melanoma, and glioblastoma [24]. CAR-T cells expressing this chimeric receptor reduced the susceptibility to tumor-induced hypofunction in various solid malignancies compared to CAR-T cells that do not carry the receptor [78]. The results from such studies and others are encouraging and point to the potential of chimeric receptors to increase the efficacy of immunotherapeutic strategies.

### Chimeric antigen receptor (CAR) T cells

**Fig. 4:**
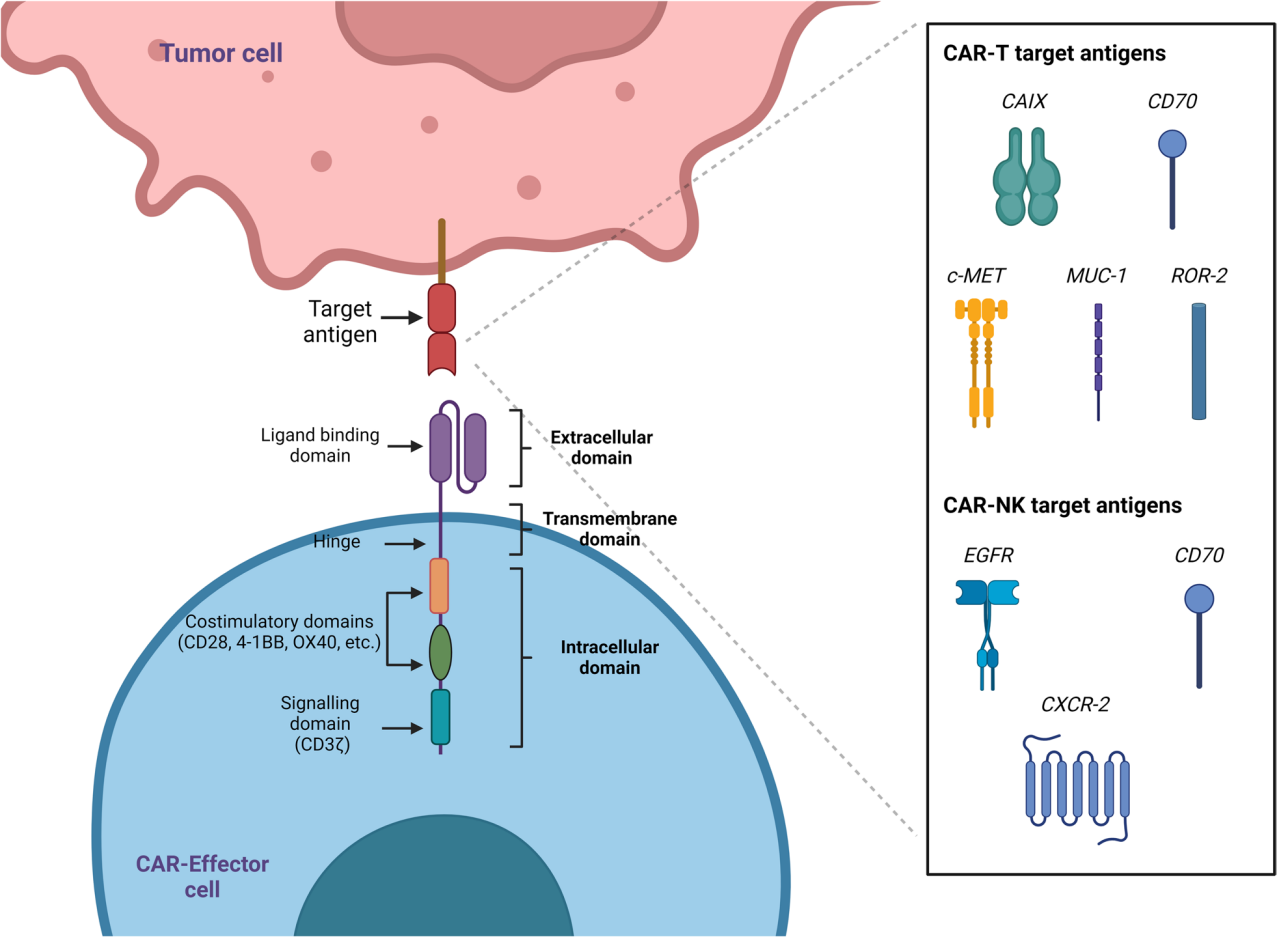
Chimeric antigen receptor (CAR) structure. The CAR consists of an extracellular-hinge region, usually based on a single chain viable fragment (scFv), linked to a transmembrane region and intracellular costimulatory (e.g., 4-1BB, CD28) and stimulatory domains (CD3z). The CAR can be expressed in different immune cells (e.g., T cells, NK cells, Macrophages) and recognize specific tumor antigens independently of the major histocompatibility complex (MHC) presentation. This image summarizes all the molecules that are currently being evaluated as potential targets for CAR in RCC: carbonic anhydrase IX (CAIX), CD70, tyrosine-protein kinase Met (c-Met), mucin-1 (MUC1), receptor orphan tyrosine kinase receptor 2 (ROR2), epidermal growth factor receptor (EGFR). Created with BioRender.com

CARs are genetically engineered receptors designed against one or more antigens and expressed on immune cells (Fig. 4). Their extracellular domain is usually an scFv capable of specifically binding antigens overexpressed at the surface of tumor cells, linked to a hinge domain (e.g., CD8, CD28, IgG1, or IgG4) and a transmembrane domain (e.g., CD28, 4-1BB or CD8), fused to one or more variable intracellular costimulatory domains (e.g., CD28, 4-1BB, or OX40 – not present in first generation CARs) and a CD3ζ activation domain, leading to full T cell activation after contact with the target antigen [27]. CAR-T cell therapy has led to significant advances in cancer cell immunotherapy, resulting in great success in treating hematological malignancies [79], with recent advances for solid tumor treatments [80], including RCC [81].

#### Lessons learned from the first CAR-T cell therapy (G250)

Carbonic anhydrases (CAs) are metalloenzymes that catalyze the reversible hydration of carbon dioxide to bicarbonate and hydrogen ions, controlling the pH of different body compartments. CAIX is a CA isoform overexpressed in hypoxic conditions and constitutively expressed in the majority of cases of the most common type of RCC, the ccRCC, due to mutations of the VHL gene, CAIX is being used as a ccRCC biomarker and has been shown to have prognostic implications [82, 83].

The first anti-CAIX CAR-T cells clinically tested for the treatment of metastatic ccRCC were CD4TM-γ expressing the first-generation CAR based on murine monoclonal antibody (mAb) G250 applied in high sequential doses in combination with IL-2. Patients showed disease progression, and liver toxicity was attributed to a specific scFv CAIX G250 attack against bile duct epithelial cells [41]. In a subsequent clinical protocol with the same CAR-T cells, the authors extended their observations to attenuate the on-target off-tumor cytotoxicity against bile duct epithelial cells, by testing the use of chimeric G250 mAb as a pre-treatment strategy. However, no effective antitumor response was obtained; but the results suggested that using a human scFv anti-CAIX and other generations of CAR vectors could enhance their efficiency [42].

#### Carbonic anhydrase IX-specific CAR-T cells alone or in association

In preclinical studies, Lo et al. tested two main constructs preclinically: a 1st generation humanized anti-CAIX scFv G36 CAR with only CD3ζ activation domain (G36-CD3ζ); and a 2nd generation G36 CAR containing the costimulatory cassette CD28 (G36-CD28ζ). The G36-CD28ζ CAR-T cells exhibited superiority compared to the first-generation equivalent CAR, with partial tumor regression observed in 67% of the cases [25]. To improve the efficacy of G36-CD28ζ against ccRCC, Suarez et al. tested armored anti-CAIX G36 CAR-T cells secreting anti-programmed cell death ligand-1 (PD-L1) IgG1 or IgG4 mAb, which target PD-L1 positive ccRCC cells and block the PD-1/PD-L1 pathway. These CAR-T cells, in a dose equivalent to 10^8^ CAR CD8 T cells/kg, were able to reverse T cell exhaustion, decreasing 30–70% expression of exhaustion markers in tumor-infiltrating lymphocytes (TILs). There was an improvement in antitumor activity, with a robust decrease in tumor weight by 50–80% compared to a parental anti-CAIX CAR-T cell [26]. The anti-CAIX G36 CAR CD28 or BBζ peripheral blood mononuclear cells (PBMCs) secreting anti-PD-L1 IgG4 were recently tested in low doses in a similar orthotopic ccRCC model, equivalent to ≅ 10^7^ CAR-T PBMCs/kg, and the CD28-based construction was superior to BBζ when immune checkpoint blockade via PD-L1 was used in combination. This treatment reduced tumor weight by 60%, avoided the occurrence of metastasis, and showed a 50% reduction in the co-expression of T cell exhaustion markers on viable TILs. The authors suggest a synergistic effect of CD28-based CARs with a PD-L1 blockade concerning the reversal of immunosuppression. No hepatotoxicity or nephrotoxicity was observed [84].

Recently, Wang et al. compared different anti-CAIX G36 CAR constructs (BBζ, 28ζ, 28BBζ) and CD4/CD8 cell compositions in an orthotopic mouse model bearing human ccRCC. The results showed that anti-CAIX G36 BBζ CAR-T cells with a CD4/CD8 ratio of 2:1 demonstrated complete tumor regression and exhibited decreased exhaustion genes revealed by single-cell RNA sequencing (scRNAseq) [28].

The combination of the TKI sunitinib with another anti-CAIX CAR-T containing a CD8α transmembrane domain and the intracellular domains of 4-1BB and CD3ζ in a subcutaneous mouse lung metastasis model of human RCC has led to the survival of all mice at the end of the experiment (day 60), with decreased tumor burden compared to anti-CAIX CAR-T cells or sunitinib alone. Sunitinib enhanced the proliferation and infiltration of CAIX-CAR-T cells, with decreased frequency of myeloid-derived suppressor cells in tumors [29]. Another study combined an oncolytic adenovirus (OAV) carrying decorin with CAIX-targeted CAR consisting of an scFv, a CD8α transmembrane domain, and 4-1BB/ CD3 zeta signaling intracellular domains. The CAIX CAR-T and OAV-Decorin (OAV-DEC) construct proved to have a significant specific killing effect on CAIX-positive RCC cells in vitro and displayed synergistic antitumor effects. In a subcutaneous xenograft model of human RCC, the combination OAV-DEC + CAIX-CAR-T reduced the tumor volume by 87%, while OAV + CAIX-CAR-T reduced the tumor volume by 54% [39].

#### CD70 targeted CAR-T cells

CD70 is a membrane protein that binds to the tumor necrosis factor receptor (TNFR) known as CD27. Hematologic malignancies and solid tumors, including about 40% of RCC cases, may constitutively express CD70 in high levels [85, 86]. Anti-CD70 CAR-T cells have shown an antitumor effect on RCC in preclinical studies leading to lysis of target cells and increased levels of IL-2, TNF-α, and IFN-γ released by CAR-T cells. Also, in the same study, the addition of the poly (ADP-ribose) polymerase (PARP) inhibitor olaparib (OLA) associated with CD70 CAR-T showed an increase in CD8 + infiltration and a better survival rate among tumor-bearing mice [30]. Another preclinical study evaluating allogeneic cells identified CD70 CAR-T binding epitopes that exhibited important antitumor activity against RCC cell lines and in a xenograft mouse model of RCC derived from patients [31]. Moreover, we developed dual-targeted anti-CAIX/CD70 CAR-T cells to enlarge the target cell population and mitigate tumor heterogeneity [87, 88].

Clinical trials are in progress to investigate the safety and efficacy of CD70-targeted CAR-T cells (NCT02830724) against several solid tumors including RCC. The food and drug administration (FDA) has granted fast-track designation to the Phase I (TRAVERSE) study to investigate the efficacy of an allogeneic CAR-T cell therapy that targets CD70 (NCT04696731). A Phase I multicenter trial (COBALT-RCC) of CRISPR-(CTX-130) in fourteen subjects with stage IV CD70 positive ccRCC, from which six presented refractory disease, has led to 8% durable CR (18+ months) and 69.2% SD. The treatment induced an acceptable safety profile, with most patients presenting low cytokine release syndrome (CRS) grades. No patients had GvHD, neurotoxicity, or hemophagocytic lymphohistiocytosis [40].

#### Other CAR-T cells for RCCs

In an orthotopic mouse model, CAR-T cells targeting tyrosine-protein kinase Met (c-Met) were presented as an option for treating pRCC. Administration of CAR-T cells induced an apparent suppression of tumor growth, and complete tumor regression was achieved in approximately 60% of the mice. In addition, the study verified the synergistic increase in therapeutic efficacy when in combination with axitinib [32]. However, the clinical study did not show an objective response to treatment (NCT01218867). Other clinical trials of c-Met CAR-T cells based on different vectors are advancing (NCT03638206).

An allogeneic CAR-T cell therapy designed to target cancer cells that express the cell surface-associated C-terminal antigen Mucin-1 (P-MUC1C-ALLO1) will be tested for safety, tolerability, and response to treatment in patients with solid cancers, including RCC (NCT05239143). Phase I/II clinical trial is investigating the therapeutic effects of CAR-T CCT 301-38 or CCT 301-59 cells in stage IV metastatic patients at different molecular targets, that RCC patients with receptor orphan tyrosine kinase receptor 2 (ROR2) receive CCT31-59 while AXL-positive patients undergo CCT 301 − 38 (NCT03393936).

### CAR-NK cells or other genetically engineered NK cells

**Fig. 5:**
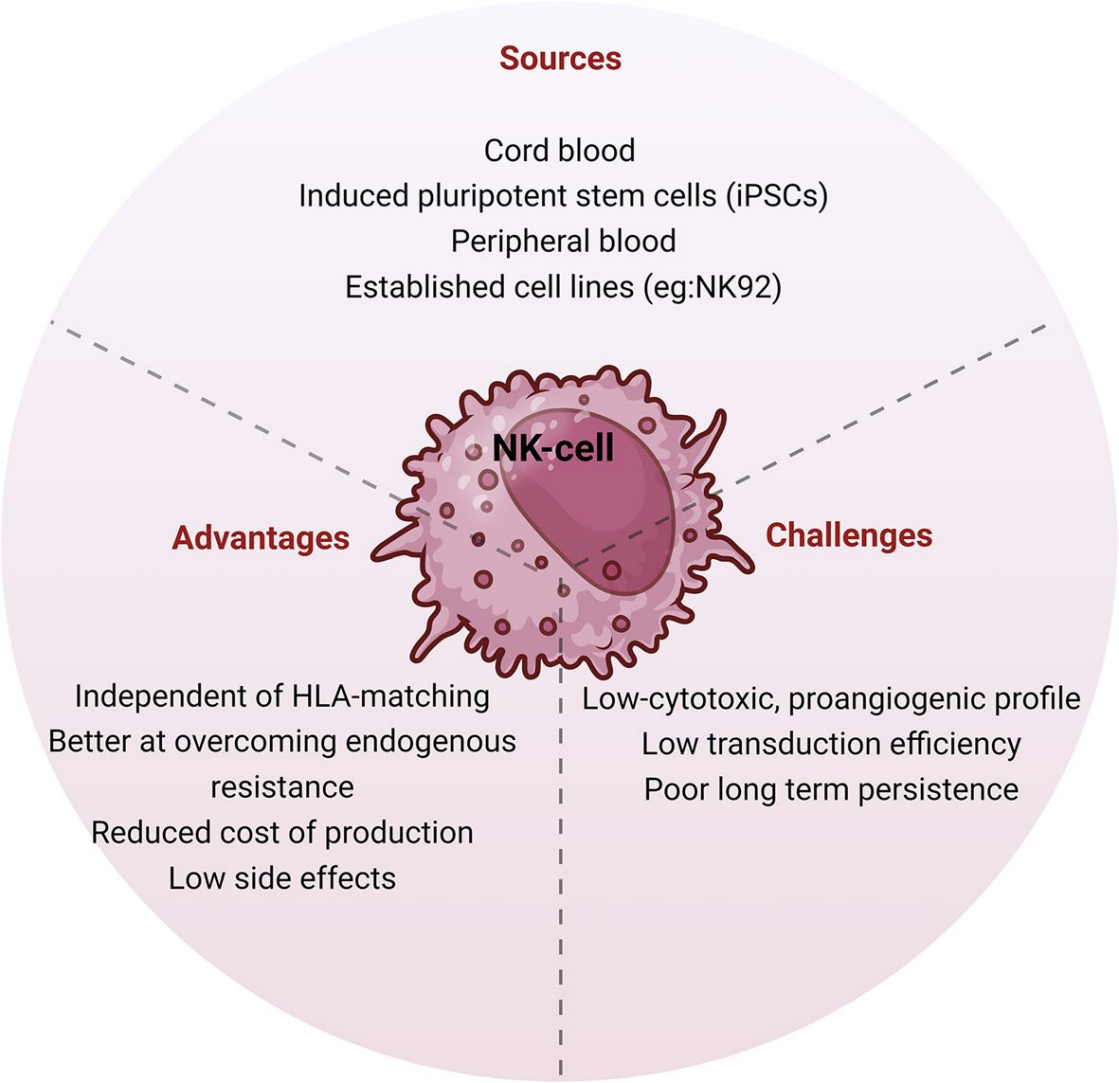
Natural killer (NK) cells for adoptive cell therapies. Description of advantages and drawbacks of NK cells used for adoptive cell therapies against RCC, showing the possible sources of these cells. Created with BioRender.com

A wide range of sources can provide NK cells, such as peripheral blood, cord blood, induced pluripotent stem cells (iPSC), and an established NK cell line (such as NK92) can also be used [89]. Allogeneic NK cells can be infused back into patients regardless of donor-patient human leukocyte antigen (HLA) type matching, being an exciting alternative to reduce the cost of CAR-based cell therapy [90–92]. The positive rationale for using CAR-NK cell therapy includes that it is less likely to cause side effects like CRS and GvHD [89, 91] and this CAR-NK can overcome endogenous resistance mechanisms in tumor cells [93]. Nevertheless, some difficulties have been reported, such as low transduction efficiency compared to T cells and poor expansion when peripheral-blood-derived NK cells are used (Fig. 5) [94]. When the source of NK cells is from umbilical cord blood, these issues appear to be minimized; however, the relative immaturity of these cells constitutes a possible disadvantage. The use of NK-92 cell lines facilitates engineering and expansion, but it faces some challenges due to safety considerations, the necessity of special cell processing [33, 94], and poor long-term persistence [94, 95].

Other challenges in CAR-NK cell therapy production include prolonging the cell survival post-infusion with significant persistence in the peripheral blood, optimizing cell cytotoxic capabilities [90], and increasing CAR-NK cell traffic to the tumor site [89]. A review of six male RCC patients between 50 and 70 years old and no previous treatment has shown that RCC can alter the classical characteristics of NK cells towards a decidua NK-like program, limiting their cytotoxic capacity and inducing angiogenesis [96], pointing out a possible new challenge to use CAR-NK therapy against RCC.

#### CAIX-specific NK92 cells

NK92 is an IL-2-dependent immortalized cell line derived from a patient with lymphoma. As such, despite the already established general safety of infusion, NK92 must be irradiated before its clinical use [34, 97]. The theoretical advantages of NK92 include its easy expansion and availability, resulting in reduced time to start treatment with lower costs [34].

CAIX-specific CAR-NK92 cells have been described as a potential killer of RCC cells in vitro and in vivo in a mouse model of human RCC. This 3rd generation CAIX-CAR includes an anti-CAIX scFv (LV5), CD8 hinge and transmembrane regions, and CD28, 4-1BB, and CD3ζ intracellular domains [34]. CAIX-specific CARNK92 at an effector: target (E:T) ratio of 30:1 induced specific cell lysis varying from 25-55% for different RCC cell lines, with an unobtrusive 10% increase when CAIX-specific CARNK92 was combined with bortezomib, a proteasome inhibitor [34]. The combination induced a very significant tumor volume decrease in a mouse model of subcutaneous human RCC [34].

#### EGFR-Specific CAR-NK92

An epidermal growth factor receptor (EGFR)-specific 3rd generation CAR was tested against EGFR+ RCC cells in vitro and in a subcutaneously human RCC-bearing mouse model. When these CAR-T cells are combined with low doses of cabozantinib – a tyrosine kinase inhibitor (TKI) that significantly decreases PD-L1 expression and increases EGFR expression in RCC cells in vitro* –* 5-fold lower tumor volume were found when compared with CAR-NK92 cells or cabozantinib treatment alone [35].

#### CD70-directed CAR-NK cells

CD70 has also been explored as a target for therapies based on NK cells. A recently opened Phase I/II clinical trial evaluates cord blood-derived NK cells expressing a CD70-targeting CAR engineered to secrete IL-15. This treatment has been performed in association with lymphodepleting chemotherapy for the treatment of advanced RCC, mesothelioma, and osteosarcoma (NCT05703854), with no published results up to November 2023.

#### NK92 expressing CXCR2

Chemokines regulate immune cell migration by binding to their corresponding chemokine receptors. Many solid tumors, including RCC, release ligands for the C-X-C motif chemokine receptor 2 (CXCR2), but NK cells in peripheral blood seem to lose CXCR2 expression [36]. For this reason, patients with solid tumors that received adoptive NK cell infusion exhibited poor migration of NK cells to the tumor. Genetically modified NK cells re-expressing CXCR2 showed an increased ability to migrate toward CXCR2-ligand-expressing tumors, with better adhesion properties and more significant killing of target cells [36]. NK cells transduced with CXCR2 showed a 2-fold increase in their migration ability to CXCR2 ligands secreted by RCC cell lines compared to NK cells transduced with NGFR [36]. Elevated numbers of tumor infiltrating NK (TINK) cells are related to a better RCC prognosis [36].

### Dendritic cell (DC) vaccination

DC vaccines work through induction and endorsement of an immune reaction to eradicate tumor cells. Autologous DCs pulsed with peptides or tumor lysate-derived proteins can stimulate the generation of cytotoxic T cells in cancer patients. Four main methods were applied to use DCs as cell-based vaccines against cancer: co-culture of DCs with isolated autologous tumor tissues, co-culture of DCs with synthetic peptides or recombinant proteins of a tumor antigen, transfection of DCs with a specific plasmid to express tumor antigens, or fusion of DCs with complete tumor cells via polyethylene glycol [98–103]. The result of a Phase I/II trial showed DCs pulsed with telomerase and surviving-derived HLA-A2 binding peptides in association with low dose IL-2 was able to promote stable disease for more than 8 weeks in 13 out of 27 patients with RCC [43].

### Other adoptive cell therapies for RCC

#### Lymphokine-activated killer (LAK) cells (NK-like T cells)

Cappuzzello et al. reported the cytotoxic effector functions of NK-resistant tumor-killing cells, termed LAK cells [104]. LAK cells constitute T and NK cells, mainly expressing NK markers such as CD3-CD56 + and NKG2D for HLA-independent killing mechanisms [105, 106]. Autologous-activated LAK cells were infused in metastatic RCC (mRCC) patients with IL-2, and the response rate reported was relatively low with specific side effects [107]. A feasibility clinical trial was then conducted on 10 mRCC patients treated using lymphokine-activated natural killer (LANAK) cells associated with IL-2. The trial yielded 3/10 PR, 4/10 CR, and 1/10 SD with immunotherapy alone, and 2/10 CR after immunotherapy plus surgery, with IL-2 toxicities observed in all treated patients [44]. This better antitumor response allow us to conclude that using well-defined effector cells like NK cells rather than a heterogenous cell population is better for effective treatment [108] Tumor reduction and clinical toxicity were not correlated with LAK cell lytic activity or dose in RCC [109]. A Phase II trial recruited 94 patients with advanced RCC and treated them with LAK cells associated with IL-2 or bolus IL-2 injection with continuous infusion. The two groups achieved objective responses (OR) of 19% and 15%, respectively [46]. In another study for advanced RCC, LAK cells were systemically administered to patients between one to three times a week, followed by a bolus injection of 5000 IU IL-2 twice daily. These cells were localized in the lung but not in the tumor tissue and used to treat pulmonary RCC metastasis. In this study, 50% of the metastatic sites, such as bone, muscle, and lymph node metastases, showed regression in 9 patients treated by arterial LAK therapy, with no severe side effects [110]. LAK cell therapy has been replaced by more specific cell-based immunotherapies [111].

#### γδ T cells (from TIL and PBMCs)

γδ T cells are a distinct subset of T cells abundant in mucosal organs that constitute less than 5% of the peripheral blood lymphocytes [112, 113]. γδ T cells are non-HLA‐restricted cytotoxic cells and play an important immune role in innate and adaptive immunity by directly recognizing and killing pathogens and activating T and B lymphocytes by releasing certain cytokines [114]. Clinical trials have reported the safety and efficacy of activated γδ T cells in patients with non small cell lung cancer (NSCLC), RCC, melanoma, and breast cancer [45, 115–117]. Vγ9Vδ2 T cell subset is the most convenient to isolate and expand, especially from human PBMCs. Many studies have shown that these γδ T cells can be genetically engineered to have superior and specific cytotoxic effects against tumors, resulting in advanced adoptive immunotherapies. Chemotherapy-resistant γδ T cells have been developed by introducing O^6^-alkylguanine DNA alkyl transferase, a DNA repair enzyme, into Vγ9Vδ2 T cells by lentiviral transduction to confer resistance to the chemotherapeutic drug temozolomide (TMZ) to treat glioblastoma. Chemotherapy-resistant γδ T cells were reported to have a superior antitumor potential in the presence of TMZ [118]. CD19-specific CAR-T cells employing ex vivo expanded Vγ9Vδ2 T cells showed cytolytic effects against CD19+ cancer cells [119]. Different approaches have been studied and reviewed to engineer γδ T cells to overcome their clonal heterogeneity for optimal functionality. T cells engineered with defined γδ T cell repertoires are the autologous αβ T cells transduced with high-affinity Vγ9Vδ2 TCRs. These engineered cells have been subjected to a clinical trial for safety and efficacy assessment [120]. Another excellent feature of Vγ9Vδ2 T cells is that the PD-1/PD-L1 axis does not abrogate their function [121]. Based on all these properties, γδ T cells can be a good immunotherapy source for immunosuppressive solid tumors such as ccRCC. In a recent study, IL-15 activated γδ T cells showed an improved cytotoxic effect in RCC patient-derived xenograft (PDX) mouse model [37]. Lee et al. have characterized CD3low Vγ9Vδ1 T cells and explored their effector and cytotoxic function in 20 treatment naive RCC tumor samples from patients suggesting them as novel therapeutic candidates for RCC treatment in high-risk patients [38].

#### Cytokine induced killer (CIK) cells

CIK is a novel strategy of cancer cell immunotherapy based on modification, manipulation, and co-opting of autologous or allogeneic primary CD3+ CD56- T cells and CD3+ CD56+ NKT cells [122–124], in which NKT cells can recognize tumor cells in a HLA-unrestricted manner [122, 125, 126]. The results of clinical trials have shown all 40 enrolled patients treated with autologous CIK cells with significantly improved overall health conditions and OR [127]. Autologous CIK cells in combination with ICIs (pembrolizumab) [128], inflammatory cytokines (IL-2) [128], and TKIs (sorafenib) [129] have exhibited synergistic effects on RCC tumors in the clinic. Moreover, co-culturing DCs and CIK cells, termed DC-CIK, have improved anti-tumor activity and proliferation of CIK cells, might resulting from the capacity of DCs to decrease Tregs [130]. The combination therapies of DC-CIKs with ICIs (pembrolizumab) [48] (NCT03190811), and TKIs (sunitinib/sorafenib) [47] in RCC clinical trails further increase therapeutic efficacy compared to the monotherapy. Combining CIK cells with a DC vaccine also has displayed more robust antitumor activity and less severe side effects in RCC treatment [131] (NCT01924156).

## Discussion

In this manuscript, we reviewed the history of cell therapy for RCC, presenting the advances and perspectives that describe a promising scenario for the use of cell therapies to treat RCC. However, several challenges must be overcome to enable safer and more effective performances of these treatments.

CAIX has recently been reborn as an exciting target for RCC cell therapy, mainly when CAR-T cells were used. Our in vitro and in vivo preclinical studies using humanized mice bearing human RCC [132] have been encouraging from the standpoint of antitumor efficacy and minimal on-target off-tumor side effects [28, 133–135]. Besides CAIX, oncofetal antigen 5T4 (with overexpression found in over 75% of RCC patient samples) [22] and the CD70 (overexpressed in about 40% of RCC patients) [86, 88, 136], are relevant targets to be further explored for the development of new cell immunotherapies for RCC management.

T cell exhaustion due to immunosuppressive factors in the TME of solid tumors, including RCC, is a substantial obstacle to improve its therapeutic efficiency. There are some prominent cell therapies in the preclinical development for RCC. Among them, we highlight the PD-1:28 CSR, which improved CAR-T cell efficiency and upgraded low-avidity T cells, blocking T cell inactivation via PD-1 to restore their effector functions and enhance tumor cytotoxicity [24]. Further development of the PD-1:28 CSR could create a first-line treatment strategy against solid malignancies that are refractory to conventional immunotherapeutic techniques. Anti-CAIX G36 CAR-T cells were also tested in a configuration capable of blocking immune checkpoint via releasing ICI in the TME.

Despite the undeniable potential of NK cells as an anti-cancer therapeutic tool and the known correlation between NK infiltration and improved survival of RCC patients [137], there are still challenges that must be surpassed to improve the efficiency of NK therapies against RCC, such as the tendency of NK cells to move to a decidua NK-like program in RCC, characterized by limited cytotoxicity and proangiogenic functions [96]. There are ongoing clinical trials with CAR-NK cell therapies for RCC treatment, such as CD70 targeted IL-15 CAR-NK cells. Also for other solid tumors, target MUC1, NKG2D ligands, and ROBO1 [89, 95]. DC-based vaccination in combination with low-dose IL-2 is a current regimen for advanced RCC.

Some recent approaches with exciting results for treating other solid tumors but underexplored in the context of RCC cellular therapies, such as CAR macrophages (CAR-M) and CAR-Treg [138]. Macrophages are abundant in solid tumors especially RCC [71, 139], and display superior capacities of tumor tissue homing compared to T cells [140]. CAR-M targeting CD19, CD22, HER-2, CD5, and carcinoembryonic antigen-related cell adhesion molecule 5 (CEACAM5) have been developed to fight against solid tumors and hematopoietic malignancies with improved tumor control and significant activation of TME [141–144]. Recently, it reported a Phase I first in human study of an anti-HER2 CAR-M in patients with HER-2 overexpressing solid tumors [145], shedding the light on translating CAR-M for RCC therapy. CAR regulatory T cells (CAR-Treg) could also be an interesting strategy to be applied in RCC treatment, especially in circumstance of immunosuppression, such as in GvHD. Anti-EGFR CAR-Treg with CD28 has shown antigen-specific infiltration as well as suppresses the function of T effector cells (Teffs) in vivo [146], providing a potential treatment for eliminating toxicities post CAR-T infusion [147]. 

## Data Availability

Not applicable, all information in this review can be found in the reference list.

## Abbreviations

5T4: Trophoblast glycoprotein
ACT: Adoptive cell therapy
CAIX: Carbonic anhydrase IX
CAR-T: Chimeric antigen receptor T cell
CAR-M: Chimeric antigen receptor Macrophage
CAR-NK: Chimeric antigen receptor natural killer cell
CAR-Treg: Chimeric antigen receptor regulatory T cell
CAs: Carbonic anhydrases
ccRCC: Clear cell renal cell carcinoma
CEA: Carcinoembryonic antigen
CEACAM5: Carcinoembryonic antigen-related cell adhesion molecule 5
CIK: Cytokine induced killer cells
CRS: Cytokine release syndrome
CsA: Camphorsulfonic acid
CSR: Chimeric switch receptor
CTLA-4: Cytotoxic T-lymphocyte associated protein 4
CXCR2: C-X-C motif chemokine receptor 2
DC: Dendritic cell
E:T: Effector: target
EGFR: Epidermal growth factor receptor
ESMO: European Society for Medical Oncology
FDA: Food and Drug Administration
FGFR: Fibroblast growth factor receptor
GvHD: Graft-versus-host disease
GvT: Graft vs tumor
HER-2: Human epidermal growth factor receptor -2
HIF: Hypoxia-inducible factor
HLA: Human leukocyte antigen
HSC: Hematopoietic stem cell
HSCT: Hematopoietic stem cell transplantation
ICI-ICI: Dual immunotherapy
ICIs: Immune checkpoint inhibitors
ICI-TKI: A combination of immunotherapy and antiangiogenic tyrosine kinase inhibitor
IL-2: Interleukin-2
IL-2Rα: Interleukin-2 receptor α
IV: Intravenous
LAK: Lymphokine-activated killer
LANAK: Lymphokine activated natural killer cell
LRRs: Leucine-rich repeats
mAb: Monoclonal antibody
MET: MET proto-oncogene
MHC: Major histocompatibility complex
mTOR: Mammalian target of rapamycin
MTX: Methotrexate
NGFR: Neural growth factor receptor
NK: CAR natural killer
NSCLC: Non-small-cell lung cancer
OAV: Oncolytic adenovirus
OAV-DEC: OAV-Decorin
OLA: Olaparib
ORR: Overall response rate
OS: Overall survival
PARP: Poly (ADP-ribose) polymerase
PBMCs: Peripheral blood mononuclear cells
PD-1: Programmed cell death receptor-1
PD-L1: PD-1 ligand
PDX: Patient-derived xenograft
PFS: Progression free survival
pRCC: Papillary renal cell carcinoma
RCC: Renal cell carcinoma
rIL-2: Recombinant interleukin-2
ROR2: Orphan tyrosine kinase receptor 2
scRNAseq: Single-cell RNA sequencing
TBI: Total body irradiation
TCF-1: T cell factor 1
TCR: T cell receptor
TILs: Tumor-infiltrating lymphocytes
TINK: Tumor infiltrating natural killer cell
TKI: Tyrosine kinase inhibitor
TME: Tumor microenvironment
TMZ: Temozolomide
TNFR: Tumor necrosis factor receptor
Tregs: Regulatory T cells
VEGF: Vascular endothelial growth factor
VEGFR: Vascular endothelial growth factor receptor
VHL: Von-Hippel-Lindau
